# Adaptations of bite force and masseter muscle thickness to high-intensity physical training in professional athletes: a comparative cross-sectional study

**DOI:** 10.1186/s13102-025-01208-0

**Published:** 2025-07-03

**Authors:** Kyu-Lim Lee, Jun-Young Sung, Soo-Bin Kim, Hee-Jin Kim

**Affiliations:** 1 Division in Anatomy and Developmental Biology, Department of Oral Biology, Human Identification Research Institute, BK21 FOUR Project, Yonsei University College of Dentistry, Seoul, 03722 South Korea; 2 Institute of Sports Medicine & Nutrition, Kwangwoon University, Seoul, 01897 South Korea; 3 Department of Oral Anatomy, Institute of Biomaterial Implant, College of Dentistry, Wonkwang University, Iksan, 54538 South Korea; 4 Department of Materials Science & Engineering, College of Engineering, Yonsei University, Seoul, 03722 South Korea

**Keywords:** Mastication, Masticatory system, Sports dentistry, Bite force, Masseter muscle, High-intensity training, Occlusal adaptation

## Abstract

**Background:**

This study investigated how prolonged high-intensity physical training influences bite force and masseter muscle thickness, which are key indicators of adaptation in the masticatory system. Understanding the relationship between these variables may provide insights into occlusal function and performance optimization in professional athletes.

**Methods:**

Thirty-four male wrestlers (Pro: 13; Npro: 21; age: 26.7 ± 9.3 years) participated in this comparative cross-sectional study. Bite force was assessed using pressure-sensitive films, and masseter muscle thickness was measured via ultrasonography. Group comparisons and asymmetry analyses were conducted using appropriate statistical methods.

**Results:**

Professional athletes exhibited significantly greater bite force (1071.7 ± 380.2 N) than non-professionals (856.9 ± 363.1 N, *p* = 0.032). Masseter muscle thickness was also higher in professionals (15.6 ± 1.5 mm right, 15.9 ± 1.4 mm left) than in non-professionals (13.6 ± 1.3 mm right, 13.8 ± 1.2 mm left, *p* < 0.01). Bite force asymmetry was more pronounced in professionals (70.1 ± 29.3 N vs. 43.1 ± 21.8 N, *p* = 0.017).

**Conclusions:**

These findings suggest that high-intensity training is associated with distinct neuromuscular adaptations in the masticatory system. Such adaptations may affect oral stability, occlusal performance, and temporomandibular joint health. The results underscore the need for individualized strategies to optimize performance and prevent occlusal imbalances in athletes engaged in intense training.

## Background

The functional demands of sports induce widespread musculoskeletal adaptation, yet the masticatory system has been relatively neglected in this context. Sports performance is influenced by various physiological and biomechanical factors, but the role of occlusal force and jaw muscle adaptation remains insufficiently explored. While musculoskeletal adaptations due to athletic training have been widely discussed, relatively few studies have addressed how occlusal function and jaw musculature may contribute to performance optimization and injury prevention. Although interest in sports-related oral health has increased, much of the existing research has concentrated on trauma prevention through the use of mouthguards, with little attention given to the functional role of the masticatory system. Consequently, the effects of repetitive loading on occlusal force and neuromuscular adaptation remain underexplored.

The masticatory system, like other skeletal muscle systems, adapts structurally and functionally in response to prolonged physical demands. Understanding changes in muscle characteristics and maximal bite force is crucial for recognizing group-specific adaptations. Professional athletes engage in high-intensity training that inevitably alters their anatomical structures according to their sport’s specific demands [1]. Such training commonly includes frequent jaw clenching, strong occlusal engagement, and occasional impacts to the maxillofacial region, all of which may contribute to specific adaptations in the masticatory system. These factors may contribute to distinct occlusal characteristics, potentially impacting neuromuscular coordination and biomechanical efficiency. Despite the growing recognition of sports dentistry, previous studies have not sufficiently examined these broader occlusal adaptations in athletes.

Bite force, directly related to mastication, is determined by the size of the jaw-lifting muscles and their interaction with the nervous, muscular, skeletal, and dental systems [2]. Recent studies have shown a close correlation between muscle thickness and muscle strength [3]. Bakke et al. reported a significant positive correlation between masseter muscle thickness and occlusal force [4]. However, data derived from professional athletes remain limited. This study aims to address this gap by analyzing bite force distribution and masseter muscle thickness in professional athletes compared to non-professional athletes.

In this study, we assessed bite force and masseter muscle characteristics to explore potential neuromuscular adaptations in athletes. Additionally, masseter muscle thickness was measured using ultrasound, an effective tool for intuitively analyzing skeletal muscle tissue thickness. These results enable a comprehensive morphological and functional assessment of the stomatognathic system, facilitate diagnosis and prognosis, and define normality standards for professional athletes and sports populations. By focusing on fundamental occlusal characteristics of athletes, this study seeks to establish a foundational understanding of how occlusal function adapts to athletic training. This study aims to contribute to sports dentistry by providing foundational data for understanding occlusal adaptation in athletes by providing essential data on occlusal adaptation, supporting the development of interdisciplinary research that integrates biomechanics, dental health, and sports performance optimization. This study aimed to compare bite force and masseter muscle thickness between professional and non-professional athletes. We hypothesized that there would be no significant differences between the groups.

## Methods

The study included 34 male wrestlers’ participants, comprising 13 professional athletes (Pro) registered with the Korean Sports and Olympic Committee and 21 non-registered athletes (non-professional athletes: Npro), including club members. In addition to the registration status as professional athletes, the Pro group consisted of individuals who engaged in training more than six times per week or for more than 14 h per week, whereas the Npro group included those who trained less than four times per week or for fewer than eight hours per week. Although the composition of training programs may vary across gyms, they generally include basic physical conditioning, resistance training aimed at enhancing muscular strength, and wrestling-specific skill training. Participants were voluntarily recruited through a public announcement, and no apparent sampling bias that could influence the study outcomes was identified. The participants had an average of 11.5 ± 5.6 years of professional training experience (range, 0.4 to 22.0 years) and a mean age of 26.7 ± 9.3 years. We visited four teams and gyms that regularly conducted training to explain the experiment and recruited those who wished to participate voluntarily. As sex is also an important factor related to maximum bite force [2], only male participants were recruited for this study.

Our recruitment process aimed to reflect the broad diversity present in professional athletes. All participants were treated equitably, with equal access to study resources and support, ensuring no bias-influenced data collection or analysis. By addressing diverse perspectives, our findings aim to contribute to more inclusive practices in sports medicine.

The exclusion criteria were as follows: (1) history of temporomandibular joint disorders, (2) ongoing orthodontic procedures, (3) significant malocclusion issues, such as an anterior open bite, and (4) missing two or more molars (excluding a third molars). Exclusion criteria were assessed based on self-reported medical history obtained through a structured questionnaire. For cases with uncertain or ambiguous information, eligibility was determined following discussion with a licensed dentist (the corresponding author), based on participants’ verbal reports and available background information. Before each step of the study, all volunteers were provided with a detailed explanation of the study’s purpose, methods, and potential risks. They were informed of their right to withdraw from the experiment at any given time. Subsequently, participants signed an informed consent form. All study procedures were approved by the Ethics Committee of YongIn University (IRB No. 2-1040966-AB-N-01-2302-HSR-293-3) and the study was conducted according to the principles of the Declaration of Helsinki.

### Bite force measurement using an occlusal force analysis system

Bite force was assessed using Dental Prescale^®^ (GC Co., Tokyo, Japan), a validated pressure-sensitive film widely used in clinical and research applications. All volunteers with normal occlusion were asked to bite down on a Dental Prescale^®^ sheet to produce the sample. The bite force (in Newtons) was measured after practicing the test twice while sitting in a natural position without supporting the head. To ensure accuracy and familiarization with the procedure, each volunteer practiced the test twice before the final measurement. The measurement was performed by placing a Dental Prescale^®^ sheet on the occlusal surface of the upper and lower jaws and biting as hard as possible for 3 s, simulating the moment of maximum power exertion during exercise. When the sheet was bitten, the microcapsules contained in the sheet burst, detecting the occlusal contact area. Occlusal software (Occlusal 709, GC, Tokyo, Japan) was used to measure the total bite force and right and left bite forces.

### Masseter muscle thickness measurement using ultrasonography

**Fig. 1:**
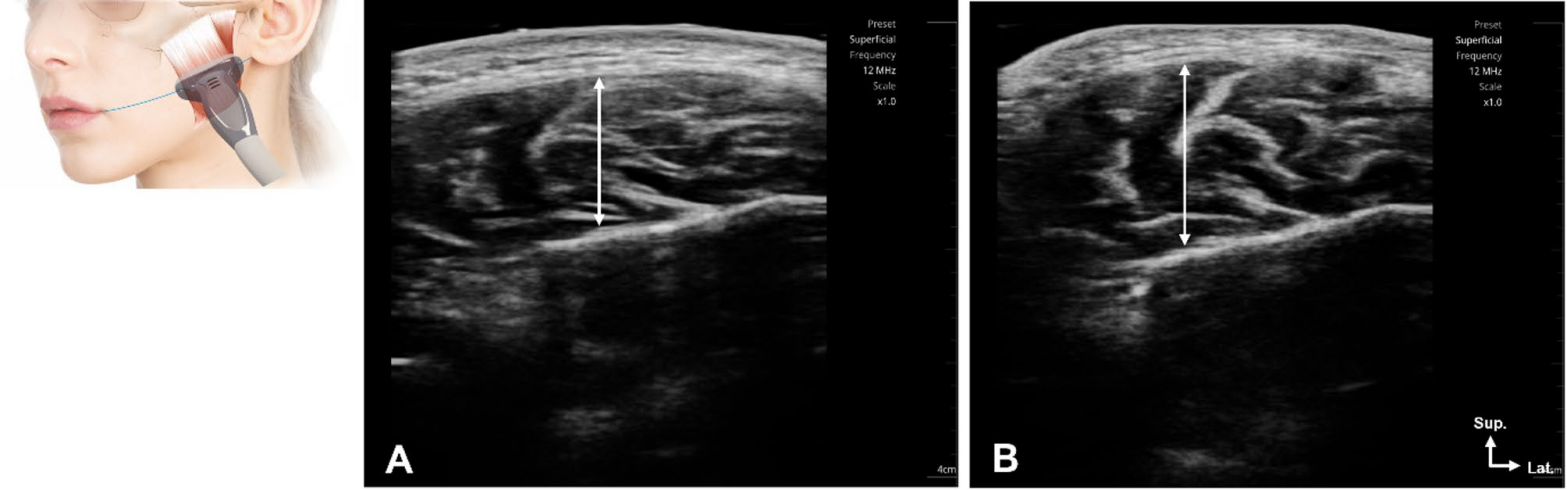
Ultrasound examination guidelines for measuring masseter muscle thickness. (**A**) Measurement in the resting state. (**B**) Measurement during maximal voluntary clenching. The probe was placed perpendicular to the skin surface at the midpoint between the zygomatic arch and the mandibular angle. Sup, superficial; Lat., lateral

The examiner requested that the subject repeat the relaxation and clenching states and then located the masseter muscle by palpating it on the skin surface. A reference line passing through the cheilion to the otobasion inferius was drawn, and measurements were taken at that location (Fig. 1). The measurement was repeated twice, and the average value of masseter muscle thickness (MMT) was derived. All ultrasound examinations were performed using a real-time 2-dimensional B-mode ultrasound device with a high-frequency linear array transducer (6 to 12 MHz; SONON 500; Healcerion Co., Ltd., Seoul, South Korea). Muscle thickness (in mm) was measured using ImageJ software (National Institutes of Health, Bethesda, MD, USA). All ultrasound and bite force assessments were conducted by two experienced examiners with extensive training in musculoskeletal ultrasonography and dental occlusion assessment. To ensure consistency, intra-rater reliability testing was conducted on a subset of 10 participants, yielding ICC values above 0.90 for both variables.

### Statistical analysis

A priori power analysis using G*Power software (effect size = 0.25; α = 0.05; power = 0.80) indicated that a minimum of 34 participants were required for sufficient statistical power [5]. Although 17 participants per group were required for accurate verification, differences occurred in the group because it induced voluntary participation (pro: n, 13; Npro: n, 21). Statistical analyses were performed using SPSS software (version 25.0, Windows). The data has been validated for normality, expressed as mean ± standard deviation (SD). A one-way analysis of variance was used to analyze the distinction between sports careers, and the Tukey test was conducted as a post-test. A paired t-test was used to check the left-right imbalance of each variable by sports careers. Pearson’s correlation analysis was conducted to analyze the relationships between the variables. Statistical significance was set at *p* < 0.05. The Shapiro–Wilk test was used to confirm the normality of the data prior to parametric analysis.

## Results

**Fig. 2:**
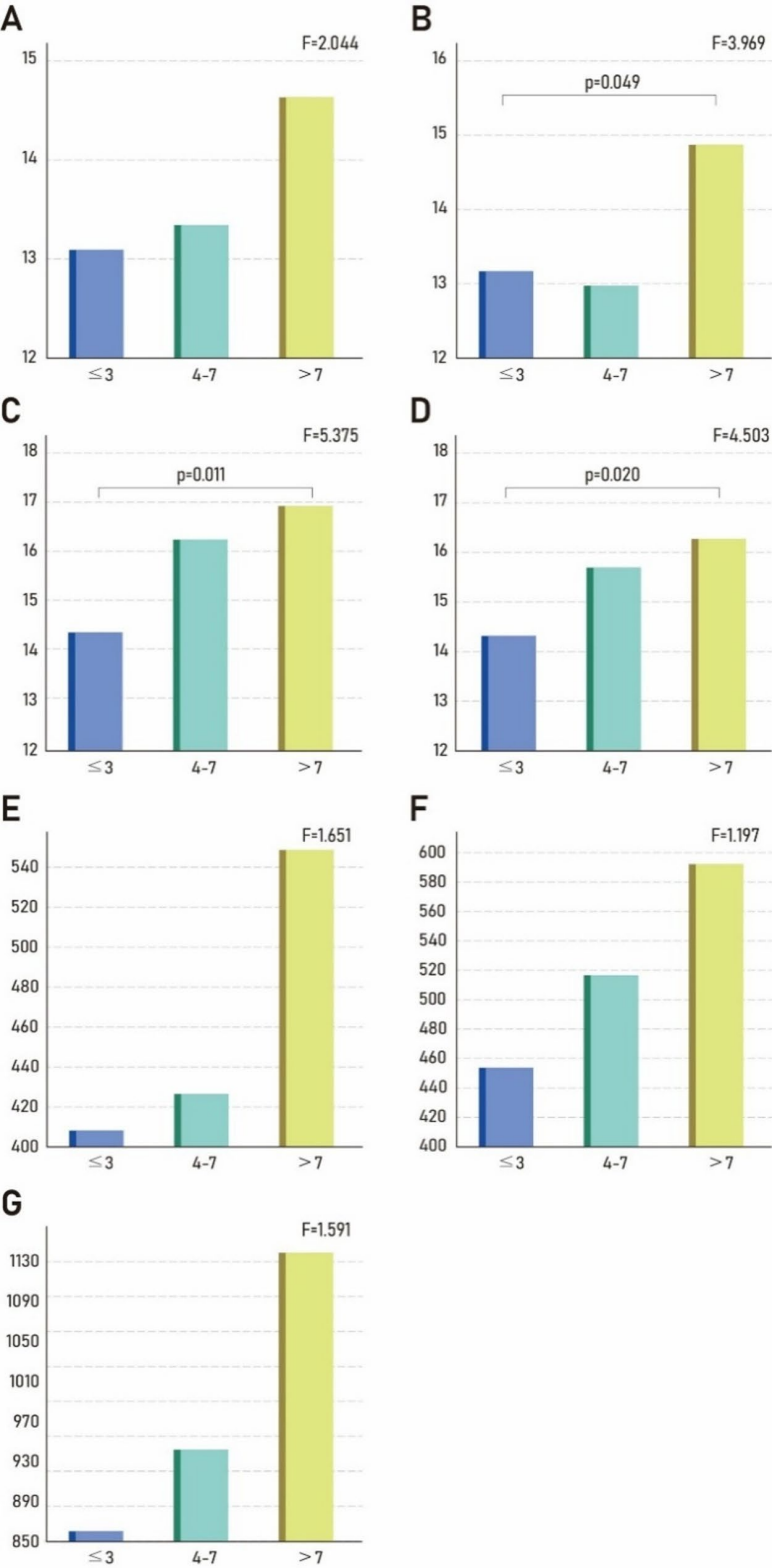
One-way ANOVA results showing differences in masseter muscle thickness and bite force based on training experience: ≤3 years, 4–7 years, and > 7 years. (**A**) Masseter thickness of the right side in resting; (**B**) Masseter thickness of the left side in resting; (**C**) Masseter thickness of the right side in clenching; (**D**) Masseter thickness of the left side in clenching; (**E**) Bite force of the right side; (**F**) Bite force of the left side; (G) Total bite force

The demographic characteristics of the participants are presented in Table 1. A one-way ANOVA of the demographic characteristics revealed a statistically significant difference in career duration among the athlete’s career (*p* < 0.001). Also, statistically significant differences were observed in the left masseter muscle thickness at rest (F = 3.969, *p* = 0.049), right masseter muscle thickness during clenching (F = 5.375, *p* = 0.011), and left masseter muscle thickness during clenching (F = 4.503, *p* = 0.020) among the athlete’s careers. While the bite force showed an increasing trend with longer training experience, the difference did not reach statistical significance (Fig. 2).

**Table 1:** The demographic characteristics of the participants

Variables		Total *n* (%)	≤ 3 years *n* (%)	4–7 years *n* (%)	> 7 years *n* (%)	F	*P*
Age (years)	≤ 19	8 (23.5)	6 (40)	2 (22.2)	0 (0)	1.056	0.360
	20–29	10 (29.4)	3 (20)	4 (44.5)	3 (30)		
	> 30	16 (47.1)	6 (40)	3 (33.3)	7 (70)		
Height (cm)	≤ 169.9	7 (20.6)	4 (26.7)	2 (45.9)	1 (10)	0.161	0.852
	170–174.9	13 (38.2)	5 (33.3)	3 (51.4)	5 (50)		
	> 175	14 (41.2)	6 (40)	4 (2.7)	4 (40)		
Weight (kg)	≤ 59.9	3 (8.8)	1 (6.7)	2 (22.2)	0 (0)	1.807	0.181
	60–69.9	4 (11.7)	3 (20)	1 (1.1)	0 (0)		
	70- 79.9	15 (44.1)	7 (46.7)	2 (22.4)	6 (60)		
	> 80	12 (35.4)	4 (26.7)	4 (44.5)	4 (40)		
Professional	Yes	13 (38.3)	0 (0)	3 (33.3)	10 (100)	26.001	**<0.001** ^***^
	No	21 (61.7)	15 (100)	6 (66.7)	0 (0)		
Total		34 (100)	15 (100)	9 (100)	10 (100)		

Significant difference, ***p < 0.001 by Tukey-test

In the non-professional athlete group (Npro group), the thicknesses of the right and left masseter muscles during rest were 13.1 ± 2.0 mm and 13.1 ± 1.8 mm, respectively. When clenched, these values increased to 14.4 ± 1.8 mm and 14.3 ± 1.4 mm, respectively. In the professional athlete group (Pro group), the masseter muscle thickness during rest was 14.1 ± 1.8 mm and 14.1 ± 1.7 mm, respectively, and when clenched, it increased to 16.8 ± 2.0 mm and 16.2 ± 1.8 mm (Table 2). The thickness of the masseter muscle at rest and the differences on both sides were similar in both groups; however, the Pro group demonstrated a greater increase in thickness during clenching.

**Table 2:** Masseter muscle thickness and bite force measurement results of beginner and expert group

Variables	RMT (mm)		CMT (mm)			BF (*N*)	
	Right	Left	Right	Left	Total	Right	Left
Pro group (*n* = 13)	14.1 ± 1.8	14.1 ± 1.7	16.8 ± 2.0	16.2 ± 1.8	1071.7 ± 380.2	500.8 ± 200.4	570.9 ± 209.0
Npro group (*n* = 21)	13.1 ± 2.0	13.1 ± 1.8	14.4 ± 1.8	14.3 ± 1.4	856.9 ± 363.1	406.9 ± 188.7	450.0 ± 203.2

Values are means ± SD, RMT, resting masseter thickness; CMT, clenching masseter thickness, BF, bite force; Npro, non-professional athletes; Pro, professional athletes

The total bite force was 856.9 ± 363.1 N in the Npro group and 1071.7 ± 380.2 N in the expert group, with the Pro group showing higher values. When measuring the bite force on the right and left sides, the Npro group’s bite force was 406.9 ± 188.7 N and 450.0 ± 203.2 N, respectively, while the Pro group’s bite force was 500.8 ± 200.4 N and 570.9 ± 209.0 N, respectively (Table 2). Significant differences in masseter muscle thickness and bite-force asymmetry were observed in the Pro group, particularly during clenching.

Table 3 presents the results of the left-right asymmetric analysis using a corresponding sample t-test based on the participants’ athletic careers. In the Npro group, no left-right asymmetry was observed between the variables. However, in the Pro group, there was a statistically significant difference in the right and left masseter thickness during clenching (*p* = 0.008) and in the right and left bite forces (*p* = 0.042).

**Table 3:** Left and right asymmetric results according to the athlete’s career

Variables		Mean ± SD	t	*p*
Pro group (*n* = 13)	RMT R-L	0.01 ± 0.88	0.05	0.480
	CMT R-L	**0.67** ± **1.03**	**2.69**	**0.008** ^**^
	BF R-L	**-70.13** ± **156.79**	**-1.84**	**0.042** ^*^
Npro group (*n* = 21)	RMT R-L	-0.01 ± 1.53	-0.04	0.485
	CMT R-L	0.05 ± 1.60	0.14	0.445
	BF R-L	-43.10 ± 152.81	-1.16	0.131

Values are means ± SD, *p < 0.05; **p < 0.01 by t-test; RMT, resting masseter thickness; CMT, clenching masseter thickness, BF, bite force; R, right; L, left; Npro, non-professional athletes; Pro, professional athletes

The correlation analysis (Table 4) showed that career duration significantly correlated with masseter muscle thickness during clenching for both the right (0.442; *p* < 0.01) and left sides (0.450; *p* < 0.01). Furthermore, masseter muscle thickness during rest on the right side was significantly correlated with that on the left side (0.794; *p* < 0.001). The strongest positive correlations among the anatomical structure variables were observed between masseter muscle thickness during clenching on the right side and resting on the right side (0.794, *p* < 0.001), between masseter muscle thickness during clenching on the left side (0.508, *p* < 0.001), and between masseter muscle thickness during resting on the right side and masseter muscle thickness during clenching on the right side (0.713, *p* < 0.001) and on the left side (0.750, *p* < 0.001). Additionally, the correlation between the total bite force and right or left bite force was significant (right: 0.927, *p* < 0.001; left: 0.937, *p* < 0.01) (Table 4).

**Table 4:** The correlation between career, masseter muscle thickness, and bite force

Variable	Career	RMT *R*	RMT L	CMT *R*	CMT L	BF *R*	BF L	BF Total
Career								
RMT-R	0.254							
RMT-L	0.338	0.794^**^						
CMT-R	0.442^**^	0.794^***^	0.713^***^					
CMT-L	0.450^**^	0.508^**^	0.750^***^	0.803^***^				
BF-R	0.269	0.093	0.145	0.077	0.184			
BF-L	0.208	0.248	0.214	0.178	0.205	0.738^***^		
BF Total	0.254	0.186	0.194	0.139	0.209	0.927^***^	0.937^**^	

Significant difference, *p < 0.05; **p < 0.01; RMT, resting masseter thickness; CMT, clenching masseter thickness, BF, bite force; R, right; L, left

## Discussion

This study aimed to compare bite force and masseter muscle thickness between professional and non-professional athletes. The main findings include: (1) significantly higher bite force in professional athletes compared to non-professionals, (2) increased masseter muscle thickness during clenching in the professional group, and (3) greater bite force asymmetry associated with longer training duration. Based on these statistically significant differences, the null hypothesis was rejected. These findings suggest that prolonged high-intensity training contributes to measurable adaptations in the masticatory system.

The total bite force in the Pro group (1071.7 ± 380.2 N) was significantly higher than in the Npro group (856.9 ± 363.1 N, *p* < 0.05), supporting the hypothesis that long-term athletic training enhances occlusal function through neuromuscular adaptation. This result is consistent with previous studies on skeletal muscles, where chronic high-load activity promotes muscle strengthening and increased force generation [3, 4].

In terms of muscle morphology, the Pro group showed greater masseter thickness (15.6 mm right, 15.9 mm left) compared to the Npro group (13.6 mm right, 13.8 mm left). This increase in muscle thickness, particularly during clenching, reflects potential structural adaptation to repeated occlusal loading, similar to changes observed in other muscles subjected to long-term physical stress [6].

Moreover, bite force asymmetry was more pronounced in professional athletes with longer training duration. This suggests that uneven load distribution during repetitive high-intensity activity may induce lateralized muscle development. Repetitive high-load activity may induce adaptive changes in the jaw joint and cervical muscles over time. This aligns with prior studies suggesting that the masticatory system, similar to other skeletal muscles, structurally adapts to functional demands [7]. Furthermore, pronounced bite force asymmetry may indicate uneven muscle loading and increased risk for temporomandibular joint issues.

A particularly noteworthy finding is the increased bite force asymmetry in professional athletes (70.1 N) compared to non-professionals (43.1 N). This asymmetry may reflect unilateral neuromuscular adaptation potentially related to sport-specific loading patterns. A phenomenon commonly observed in asymmetric sports such as tennis, golf, and combat sports. Over time, these adaptations may influence temporomandibular function and occlusal balance, necessitating further investigation into long-term effects. This suggests that sport-specific training may induce an uneven loading pattern in the masticatory system, potentially because of habitual unilateral jaw engagement. Asymmetry in muscle development is widely documented in sports that involve repetitive unilateral movements, such as tennis, golf, and combat sports [7, 8]. While some degree of asymmetry may be a natural consequence of sport-specific neuromuscular adaptations, excessive imbalance could contribute to malocclusion, temporomandibular joint (TMJ) dysfunction, or uneven craniofacial loading over time [9, 10]. Further longitudinal studies are required to determine whether occlusal asymmetry in athletes progresses with continued training and whether it has clinical implications for long-term musculoskeletal health.

Despite increasing recognition of sports-related oral health, most research has focused on trauma prevention rather than functional adaptations of the masticatory system [11, 12]. While mouthguards are widely studied for their role in preventing maxillofacial injuries [13, 14], little attention has been given to the long-term effects of high-intensity training on occlusal biomechanics and neuromuscular function. These findings align with recent literature suggesting that occlusal condition and oral structures can influence muscle activity and athletic performance, supporting a growing recognition of the masticatory system’s functional relevance in sports contexts [15–17]. Our study shifts the focus from passive protection to active adaptation, emphasizing the importance of understanding how occlusal forces evolve in response to sustained athletic training. Given that muscle activation patterns and force distribution play a role in both injury prevention and performance optimization, investigating occlusal adaptations could provide valuable insights for both sports science and sports medicine.

These findings highlight the need for a more integrated approach to understanding the relationship between occlusal function, muscle adaptation, and athletic performance. Given the growing emphasis on personalized sports medicine, future research should examine how different training modalities influence occlusal biomechanics, particularly in sports that demand high occlusal forces or jaw stabilization [18–20]. Additionally, investigating whether occlusal asymmetry progresses with continued training, training duration, intensity, and sport-specific movement patterns that influence loading mechanisms, and its potential implications for temporomandibular joint health will be crucial in developing preventive strategies for athletes. Moreover, further studies are needed to assess whether bite force asymmetry in athletes affects neuromuscular coordination, TMJ health, or overall performance efficiency [9, 10, 21]. Developing evidence-based strategies to optimize occlusal balance and prevent maladaptive changes could be beneficial for both athletes and sports dentistry applications.

By establishing fundamental reference data on occlusal force and masseter muscle adaptation in professional athletes, this study provides a foundation for future interdisciplinary research. While this research primarily focuses on foundational data collection, it underscores the importance of considering occlusal function as part of an athlete’s overall biomechanical profile [3, 4]. Future investigations should expand on these findings by incorporating sport-specific analyses and longitudinal studies to refine our understanding of the interplay between high-intensity training, occlusal adaptation, and athletic performance [19, 22]. These insights may ultimately contribute to the development of targeted interventions for optimizing occlusal function in high-performance athletes, further integrating sports dentistry into modern sports science. Although occlusal biomechanics in athletes has received limited attention outside of traditional dental perspectives, exploring its role within the context of sports science may provide valuable insights. Bridging this gap could not only refine our understanding of occlusal adaptation but also support advancements in personalized protective equipment, such as custom mouthguards and headgear, tailored to sport-specific demands. From a practical standpoint, coaches and sports health professionals are encouraged to monitor occlusal function and bite asymmetry in athletes. Introducing individualized preventive strategies, such as customized occlusal devices, jaw training programs, and balanced muscle conditioning, could mitigate the risk of temporomandibular dysfunction and improve athletic performance.

While this study provides valuable insights into the effects of high-intensity training on the masticatory system, certain limitations should be acknowledged. The sample size, though statistically sufficient, could be expanded in future studies to enhance generalizability. This study also primarily focused on combat sports athletes, and further investigations across various disciplines would provide a more comprehensive understanding of occlusal adaptations. Additionally, as a cross-sectional study, it cannot infer causal relationships, and longitudinal designs are warranted to clarify temporal changes. Finally, although the Dental Prescale^®^ provides objective measurements, it reflects only static bite force at a single time point and does not capture the dynamic functional loading during athletic activity.

## Conclusion

This study suggests that high-intensity physical activity may be associated with structural and functional characteristics of the masticatory system. Professional athletes exhibited greater occlusal force and masseter muscle thickness, indicating that professional athletes exhibit increased bite force capacity and muscle development, which may be associated with prolonged training. Additionally, the increased bite force asymmetry in athletes suggests that sport-specific training may contribute to uneven loading patterns, which could have implications for long-term oral health and musculoskeletal balance.

While previous research has focused on trauma prevention, our results suggest that understanding occlusal adaptations is equally important for optimizing athletic performance and neuromuscular stability. These findings highlight the need to consider occlusal function in sports science and injury prevention.

This study is among the first to suggest that occlusal biomechanics and masticatory muscle symmetry should be considered in the context of optimizing athletic performance and preventing functional imbalance. Future studies should investigate sport-specific occlusal adaptations, the long-term impact of asymmetry, and potential interventions to optimize occlusal balance in athletes. This research lays the groundwork for integrating occlusal biomechanics into sports medicine, contributing to performance enhancement and oral health management in high-performance athletes.

## Data Availability

The datasets used and analyzed during the current study are available from the corresponding author upon reasonable request. This statement is also provided in the manuscript submission system under “Data availability.”

## Abbreviations

ANOVA: Analysis of Variance
BF: Bite Force
CMT: Clenching Masseter Thickness
MMT: Masseter Muscle Thickness
Npro: Non-Professional Athletes
Pro: Professional Athletes
RMT: Resting Masseter Thickness
TMJ: Temporomandibular Joint
IRB: Institutional Review Board
NRF: National Research Foundation
SD: Standard Deviation
SPSS: Statistical Package for the Social Sciences
